# Pharmacological conditioning for juvenile idiopathic arthritis: a potential solution to reduce methotrexate intolerance

**DOI:** 10.1186/s12969-020-0407-5

**Published:** 2020-02-07

**Authors:** Rosanne M. Smits, Dieuwke S. Veldhuijzen, Henriet van Middendorp, Petra C. E. Hissink Muller, Wineke Armbrust, Elizabeth Legger, Nico M. Wulffraat, Andrea W. M. Evers

**Affiliations:** 1 Health, Medical and Neuropsychology unit, Leiden University, Leiden, P.O. Box 9500, The Netherlands; 2 Leiden Institute for Brain and Cognition, Leiden, P.O. Box 9600, The Netherlands; 3 Department Pediatric Rheumatology and Immunology, University Medical Center Utrecht, Utrecht, P.O. Box 85090, The Netherlands; 4 Department Pediatric Rheumatology and Immunology, Leiden University Medical Center, Leiden, P.O. Box 9600, The Netherlands; 5 Department Pediatric Rheumatology and Immunology, University Medical Centre Groningen, Groningen, P.O. Box 30.001, The Netherlands; 6 Department of Psychiatry, Leiden University Medical Center, Leiden, P.O. Box 9600, The Netherlands

**Keywords:** Juvenile idiopathic arthritis, Methotrexate intolerance, Side effects, Pharmacological conditioning, Conditioned immune suppression

## Abstract

**Background:**

Methotrexate (MTX) therapy has proven to be a successful and safe treatment for Juvenile Idiopathic Arthritis (JIA). Despite the high efficacy rates of MTX, treatment outcomes are often complicated by burdensome gastro-intestinal side effects. Intolerance rates for MTX in children are high (approximately 50%) and thus far no conclusive effective treatment strategies to control for side effects have been found. To address this need, this article proposes an innovative research approach based on pharmacological conditioning, to reduce MTX intolerance.

**Presentation of the hypothesis:**

A collaboration between medical psychologists, pediatric rheumatologists, pharmacologists and patient groups was set up to develop an innovative research design that may be implemented to study potential improved control of side effects in JIA, by making use of the psychobiological principles of pharmacological conditioning. In pharmacological conditioning designs, learned positive associations from drug therapies (conditioning effects) are integrated in regular treatment regimens to maximize treatment outcomes. Medication regimens with immunosuppressant drugs that made use of pharmacological conditioning principles have been shown to lead to optimized therapeutic effects with reduced drug dosing, which might ultimately cause a reduction in side effects.

**Testing the hypothesis:**

This research design is tailored to serve the needs of the JIA patient group. We developed a research design in collaboration with an interdisciplinary research group consisting of patient representatives, pediatric rheumatologists, pharmacologists, and medical psychologists.

**Implications of the hypothesis:**

Based on previous experimental and clinical findings of pharmacological conditioning with immune responses, we propose that the JIA patient group is particularly suited to benefit from a pharmacological conditioning design. Moreover, findings from this study may potentially also be promising for other patient groups that endure long-lasting drug therapies.

## Background

JIA is a childhood rheumatic disorder for which methotrexate (MTX) is the drug of choice, after the administration of nonsteroidal anti-inflammatory drugs and intra-articular corticosteroid injections, MTX is regarded as a safe drug with a high efficacy rate up to 70% of the patients reaching remission (1–5). However, MTX therapy is hampered by side effects such as nausea and vomiting, also known as MTX intolerance, which is one of the leading causes of discontinuation or reduction of MTX therapy and as a result causes a delay in reaching remission (6, 7). Aside from pharmacological side effects that occur after MTX intake, patients also report psychological side effects prior to MTX intake and when thinking of MTX, known as anticipatory and associative complaints (8). These complaints, for example anticipatory nausea, frequently occur and significantly contribute to the burden the patient experiences (9). Since the development of a clinical measure that determines the severity of MTX intolerance, the Methotrexate Intolerance Severity Scale (MISS), different cohort studies demonstrated that approximately 50% of the patients suffer from MTX intolerance (6–8, 10). Moreover, the MISS brought forth new insights in the development of MTX intolerance, indicating that the majority of patients develop intolerance after 6 to 12 months (7). To date, strategies that focus on the reduction of MTX side effects, consisting of anti-emetic therapy, changing the route of administration, and dose reduction, have unfortunately shown inconclusive results. However, these strategies often focus on pharmacological side effects and overlook the important psychological component in MTX intolerance (8, 11–16). In order to optimally benefit from MTX therapy, the urgent question arises how MTX intolerance can be overcome for the psychological components, including associative and anticipatory processes, of MTX treatment. In this Hypothesis article, we propose a novel approach that holds promise in reducing side effects and potentially also in optimizing treatment effects, which is known as pharmacological conditioning.

### Presentation of the hypothesis

**Fig. 1:**
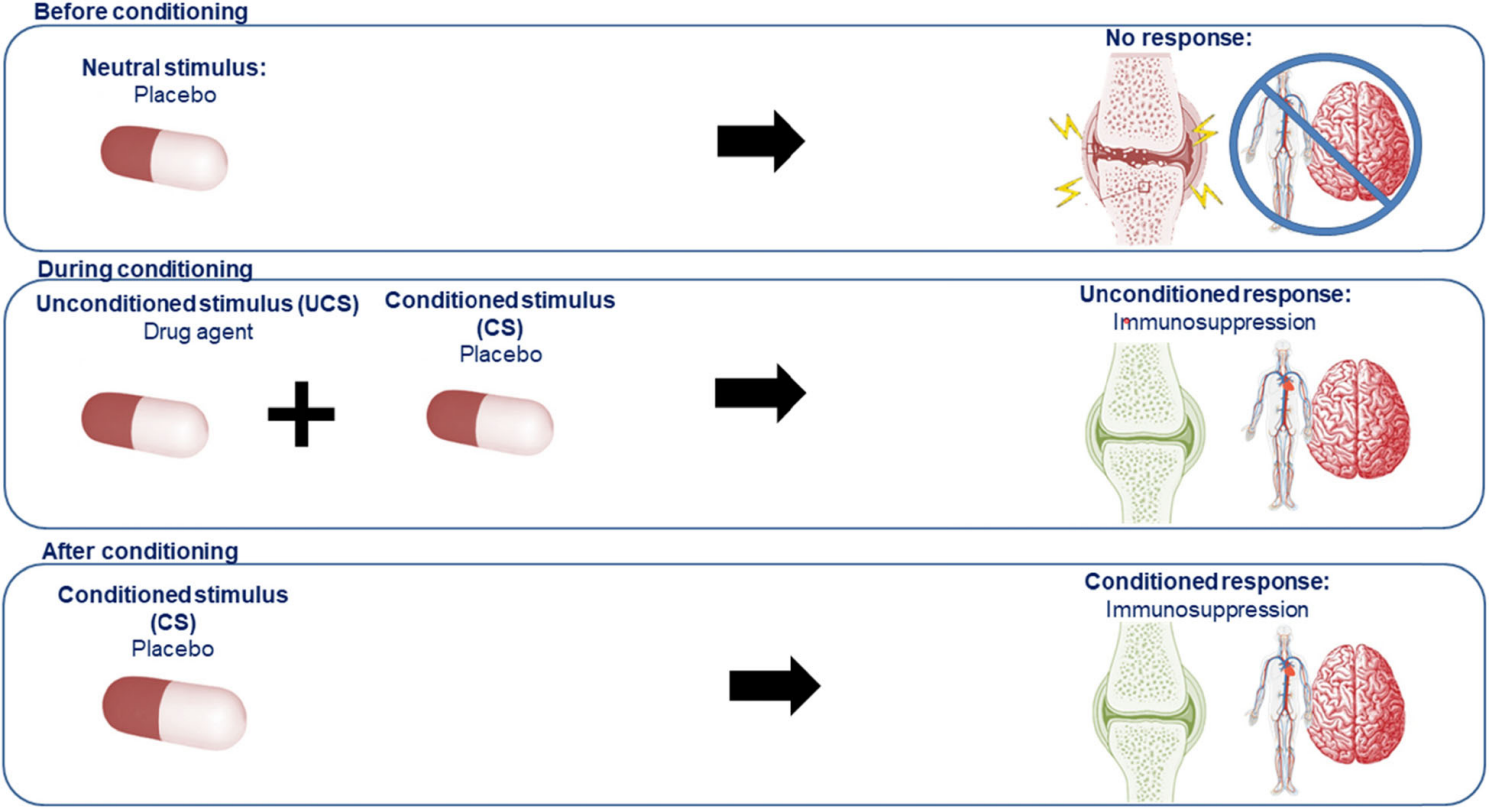
Schematic representation of conditioned immunosuppression

Behavioral learning theories, in particular classical conditioning, explain how physiological responses arise from learned associations, also known as conditioned responses. Classical conditioning was initially proposed by Ivan Pavlov and states that physiological responses can be triggered by a learned association between a stimulus and a response. Pavlov showed that when a biologically salient stimulus (the unconditioned stimulus, UCS for example food) is repeatedly paired with an initial neutral stimulus (the to-be conditioned stimulus, CS, for example a bell), a conditioned response (CR, for example the salivary response) can be triggered by the CS alone after the association has been formed (see Fig. 1) (17). Conditioned responses can manifest in both negative or positive physiological responses. For example, anticipatory nausea is an example of a conditioned response associated with MTX-related nausea, which negatively impacts health. However, positive physiological conditioned responses can also be formed during frequent and long-lasting drug therapies and simulate the initial drug effect. Research has shown that the intake of a drug can lead to a physiological response and that this learned effect can be evoked by a placebo (an inert inactive medication) (18–20). These conditioning principles have been extensively studied in the experimental field with various types of drug agents serving as the UCS (19, 21–32), including immunological agents. More recently, conditioning principles have been employed in RCTs to relieve side effects or improve treatment efficacy (18, 20). It is therefore important to explore how learning principles can be integrated in drug therapies, which will be discussed further below (17, 31).

### Pharmacological conditioning in experimental trials

Since the 1970’s, experimental conditioning trials have been developed to investigate the potential to condition pharmacological effects with immune responses. One of the initial conditioning trials conducted in rats demonstrated the ability to condition an immunological response using the immunosuppressant cyclophosphamide as a UCS, paired with saccharine (sugar water) as a CS (21). The study findings showed that the administration of saccharine at a later time resulted in a similar immunosuppressant response as cyclophosphamide, successfully demonstrating the effects of a CR (21). Subsequently, more conditioning trials followed with the immunosuppressant drug cyclosporin A and the term *learned immune response* was introduced (19, 23, 25–28, 31). In pharmacological conditioning trials, the magnitude of the CR is often measured on an immunological level, for example by a significant reduction of interleukin (IL)-2 and IFN-γ, mimicking the initial drug effects of cyclosporin A (19, 23, 25, 26, 32). More recently, conditioning effects have been found with different types of drug agents, indicating that a CR can be learned through different pathways and systems involved in the initial drug effect (23, 26). For example, in pain studies CRs were demonstrated with the opioid agonist morphine hydrochloride or the nonopioid ketorolac tromethamine, in allergy studies with the H1-receptor antagonist desloratidine (21), and in neuroendocrine studies with adrenaline, insulin, dexamethasone, glucose, IFN-β-1a or sumatriptan (30).

### Translating pharmacological conditioning to the clinical context

In the last decade, experimental findings from conditioning trials have been translated to clinical trials in humans and have shown promising results for different patient groups (18, 33–36). These studies utilized pharmacological conditioning for different objectives. One important objective has been to add identical looking placebos as CS, to evoke CRs in order to maximize therapeutic outcomes. Recently, a clinical study demonstrated that adding placebos as ‘dose extenders’ successfully enhanced therapeutic effects of cyclosporin A in renal transplant patients (34). Another important objective of pharmacological conditioning has been to control for side effects. This approach may be particularly relevant for the JIA patient group, because of the possibility of dose reduction while maintaining treatment efficacy. A double blind placebo-controlled RCT with patients suffering from psoriasis demonstrated similar therapeutic effects of corticosteroids combined with conditioning principles to treat cutaneous lesions with a 25% dose reduction (18). This study used a variable reinforcement schedule in which full doses of medication were intermittently replaced by lower doses combined with placebos to evoke a CR. Furthermore, these effects were not found in the dose control group that was administered the same dose. In dose control groups, the same cumulative amount of drug dosing as the conditioning group is administered but without the use of variable reinforcement principles (18). Effects of pharmacological conditioning have been demonstrated in children as well. In a study where children with ADHD received 50–75% reduced dosing of mixed amphetamine salts, a significant reduction in side effects was reported compared to the 100% dosing group, while maintaining similar therapeutic results (see Appendix 1 for an example of a medication schedule using variable reinforcement) (35). These findings hold great potential for the integration of pharmacological conditioning principles in various populations and for various drug effects, including younger populations.

### Testing the hypothesis

**Fig. 2:**
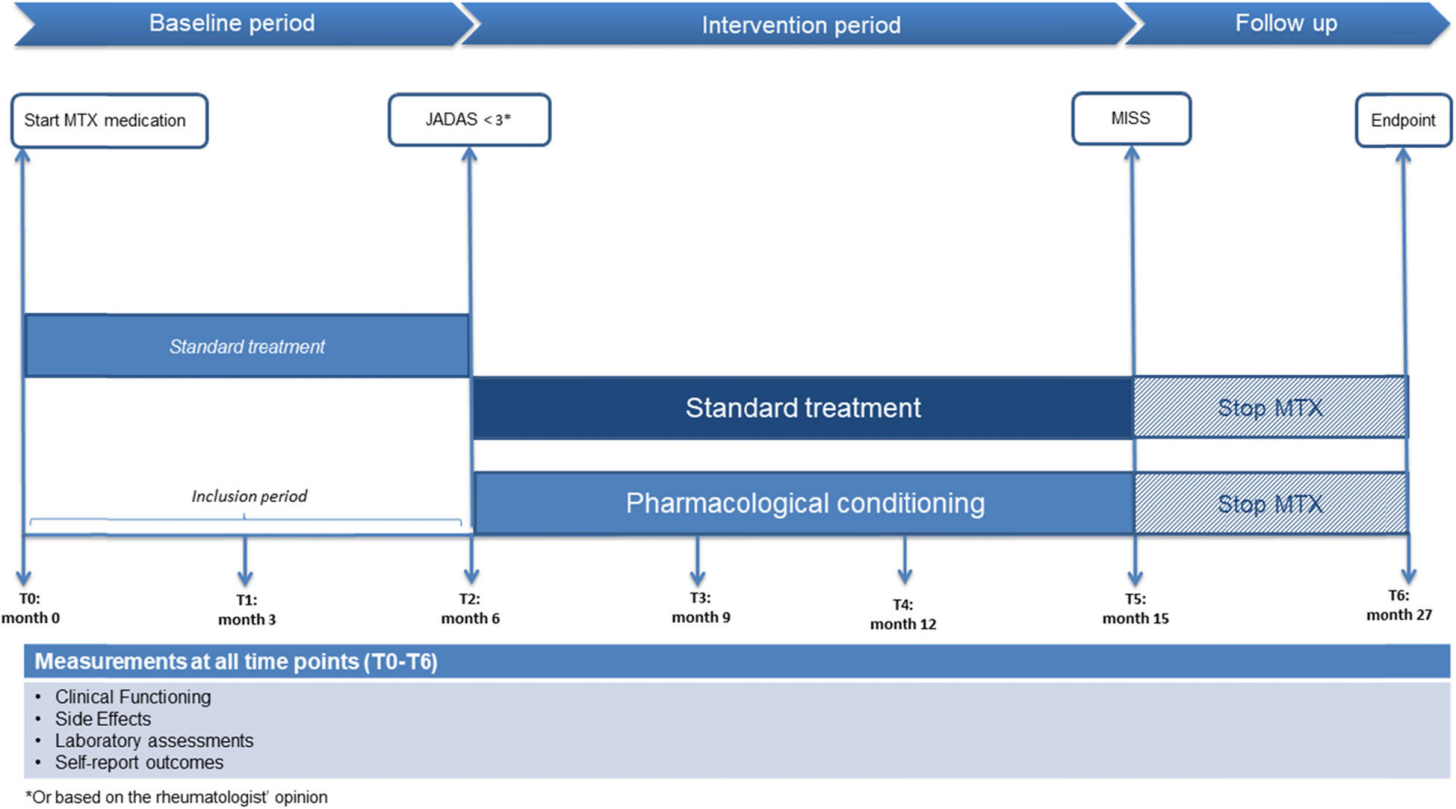
An overview of the hypothesized trial design. The clinical study design closely follows current pharmacological treatment recommendations. Baseline period: Patients diagnosed with JIA and eligible for stable standard pharmacological treatment (12,5 mg/m^2^–15 mg/m^2^) will start with MTX. Intervention period: Patients who complete the baseline period without protocol violations continue to the second phase of the study and will be randomized double-blind to one of the following groups: control group (standardized stable treatment dosages of MTX as a continuation of the baseline period for 9 months) or pharmacological conditioning group (variable doses of MTX interspersed with placebos for 9 months). Primary outcome (MISS) will be measured at 15 months (T5). This study will be closed with an end-of-study one year after the intervention period (T6). Flare-ups and side effects will be monitored during clinical visitations.

Considering the extensive field of pharmacological conditioning in experimental animal and human trials, and the possibility that different patient groups may benefit from these applications, we believe the time is now to capitalize on this treatment method. We therefore developed a novel design based on pharmacological conditioning for JIA patients, which can be found in Fig. 2. This study design is based on the assumption that conditioning effects are formed during a baseline acquisition period and are evoked in the intervention period by making use of placebo-controlled dose reduction. Participants from age 4 to 17 (at the time of JIA diagnosis) with all JIA subtypes (with the exception of systemic JIA) could be included. During the baseline period, stable doses of oral MTX should be administered (12,5–15 mg/m^2^/week) allowing the formation of a positive association between the drug and its positive therapeutic effects. The study should only use oral MTX, because it is important that the conditioned stimulus is the same for all participants to allow a comparison between groups. The baseline period ends after remission is achieved (based on a JADAS score of ≤3 or on the assessment of the pediatric rheumatologist) with a maximum duration of 6 months (7). After the baseline period, patients can be randomized to the intervention or control group where allocation should be stratified by weight (e.g., below or above 30 kg) to ensure for numerical equality. During the intervention period, conditioned responses can then be utilized by integrating pharmacological conditioning principles through a variable reinforcement schedule in which intermittent standard MTX doses and lower MTX doses supplemented with placebos are provided to evoke a CR in the low dose weeks (see Appendix 1 for an example of a medication schedule). Similar to previous conditioning trials, we propose that this reduced drug dosing may ultimately lead to lower MTX intolerance, while maintaining therapeutic efficacy (18, 35). During the development of this design, different stakeholders involved in JIA treatment were consulted to discuss a possible design for pharmacological conditioning (i.e. pediatric rheumatologists, pharmacologists, medical psychologists and patient groups). Overall, and specifically expressed by patients, the main priority was to reduce MTX intolerance. For this reason, the primary aim should preferably focus on MTX intolerance with MISS as the primary outcome. This study would therefore be powered to find a difference in MISS scores between the experimental and the control group after the intervention period (with lower MISS scores in the experimental compared to the control group). For a secondary analysis, focus can be on the effects of conditioning on an immunological level, for example in clinical measures (e.g. erythrocyte sedimentation rate and C-reactive protein level), cytokines (IL-1β, IL-6, IL-8, Interferon-γ (IFN-γ), and Tumour Necrosis Factor-α (TNF-α), MRP8/14 serum (to compare flare risks for both groups) and polyglutamates in erythrocytes (to compare intracellular buildup of MTX in both groups, which may be mimicked by the conditioned response in de intervention group). Several factors were taken into account while conceptualizing the current design. One important consideration was a dose control group. For methodological purposes, pharmacological conditioning designs often integrate a dose control group to expose direct effects of conditioning. In clinical studies with vulnerable patient groups, like children with JIA, a dose control group would be unwanted as this may cause for higher flare-up risks. However, cytokine levels and other markers for the level inflammation make it possible to investigate the effects of conditioning on an immunological level. Another consideration could be to first implement a conditioning design in an adult population, for example in patients with rheumatoid arthritis. However, in contrast to the JIA population, side effects in the adult population are less common and therefore this population may be suboptimal to test this design first (37). Nonetheless, a currently ongoing pharmacological conditioning study with MTX and RA patients did indicate the potential for conditioning with MTX (38). Finally, an important consideration is whether conditioning of therapeutic effects may also lead to conditioning of the unwanted side effects. Because conditioning plays a large role in the proposed design, this may also pose as a concern. However, recommendations that focused specifically on the psychological constructs of side effects (nocebo effects) stress the importance of managing patient expectations, considering patient–physician communication and relationships, positive framing of treatment information and emphasizing therapeutic effects, which can be employed by focusing on the positive conditioning effects in this study design (39, 40). To optimally integrate conditioning principles in drug regimens, it would therefore be important to explain the potential of pharmacological conditioning by primarily focusing on the therapeutic effects of MTX (40). Moreover, previous trials that made use of pharmacological conditioning showed a clinically meaningful reduction of side effects (35, 41, 42). Nevertheless, it is of utmost importance to closely monitor side effects during the whole duration of treatment.

### Implications of the hypothesis

To conclude, converging evidence has demonstrated the potential to condition immune responses and the possibility to integrate this in treatment designs. Pharmacological conditioning principles show potential to address an important problem in JIA treatment: it can be used to optimize MTX therapy by dose reduction and therefore possibly lower side effects while maintaining therapeutic efficacy. Based on the difficulties that the JIA patient group faces, this group may particularly benefit from this proposed study design. Ultimately, implementing a pharmacological conditioning design would not only have implications for the JIA patient group, but may also show potential for other patient groups that endure long-lasting drug therapies.

## Data Availability

Not applicable.

## Appendix

Appendix 1: An example MTX schedule for the intervention (conditioning) group and control group. The medication schedules that were developed by our group start from 7,5 mg up to 20 mg. Depending on body surface area we made schedules according to the ratio of placebos and the dose of MTX. For example, a child that receives 15 mg will receive 2 MTX pills (5 mg) during the low dose week interspersed with placebos (3:1 ratio). During baseline period (week 1 t/m 26) patients receive the same amount as the control group

**Table:**

Week	Conditioning group	Control group
*27*	15 mg MTX/wk	15 mg MTX/wk
*28*	15 mg MTX/wk	15 mg MTX/wk
*29*	5 mg MTX/wk	15 mg MTX/wk
*30*	15 mg MTX/wk	15 mg MTX/wk
*31*	15 mg MTX/wk	15 mg MTX/wk
*32*	5 mg MTX/wk	15 mg MTX/wk
*33*	15 mg MTX/wk	15 mg MTX/wk
*34*	5 mg MTX/wk	15 mg MTX/wk
*35*	15 mg MTX/wk	15 mg MTX/wk
*36*	5 mg MTX/wk	15 mg MTX/wk
*37*	5 mg MTX/wk	15 mg MTX/wk
*38*	15 mg MTX/wk	15 mg MTX/wk
*39*	5 mg MTX/wk	15 mg MTX/wk
*40*	5 mg MTX/wk	15 mg MTX/wk
*41*	15 mg MTX/wk	15 mg MTX/wk
*42*	5 mg MTX/wk	15 mg MTX/wk
*43*	5 mg MTX/wk	15 mg MTX/wk
*44*	15 mg MTX/wk	15 mg MTX/wk
*45*	5 mg MTX/wk	15 mg MTX/wk
*46*	5 mg MTX/wk	15 mg MTX/wk
*47*	15 mg MTX/wk	15 mg MTX/wk
*48*	5 mg MTX/wk	15 mg MTX/wk
*49*	5 mg MTX/wk	15 mg MTX/wk
*50*	5 mg MTX/wk	15 mg MTX/wk
*51*	15 mg MTX/wk	15 mg MTX/wk
*52*	5 mg MTX/wk	15 mg MTX/wk
*53*	5 mg MTX/wk	15 mg MTX/wk
*54*	5 mg MTX/wk	15 mg MTX/wk
*55*	15 mg MTX/wk	15 mg MTX/wk
*56*	5 mg MTX/wk	15 mg MTX/wk
*57*	5 mg MTX/wk	15 mg MTX/wk
*58*	5 mg MTX/wk	15 mg MTX/wk
*59*	15 mg MTX/wk	15 mg MTX/wk
*60*	5 mg MTX/wk	15 mg MTX/wk
*61*	5 mg MTX/wk	15 mg MTX/wk
*62*	15 mg MTX/wk	15 mg MTX/wk
*63*	5 mg MTX/wk	15 mg MTX/wk
*64*	5 mg MTX/wk	15 mg MTX/wk
*65*	5 mg MTX/wk	15 mg MTX/wk
**Cumulative dose**(%)	**335 mg** (57%)	**585 mg** (100%)

## Abbreviations

CR: Conditioned response
CS: Conditioned stimulus
IFN-β-γ: Interferon-β-γ
IFN-γ: Interferon-γ
IL: Interleukin
JIA: Juvenile idiopathic arthritis
MISS: Methotrexate intolerance severity score
MTX: Methotrexate
RA: Rheumatoid arthritis
UCS: Unconditioned stimulus
